# Discriminatory health care reported by seriously ill LGBTQ+ persons and partners: Project Respect

**DOI:** 10.1017/S1478951524001913

**Published:** 2025-04-25

**Authors:** Cathy Berkman, Gary L. Stein, William E. Rosa, Kimberly D. Acquaviva, David Godfrey, Imani Woody, Shail Maingi, christian gonzález-rivera, Carey Candrian, Sean O’Mahony, Noelle Marie Javier

**Affiliations:** 1 Graduate School of Social Service, Fordham University, New York, NY, USA; 2 Wurzweiler School of Social Work, Yeshiva University, New York, NY, USA; 3 Department of Psychiatry & Behavioral Sciences, Memorial Sloan Kettering Cancer Center, New York, NY, USA; 4 School of Nursing, University of Virginia, Charlottesville, VA, USA; 5 Commission on Law and Aging, American Bar Association, Washington, DC, USA (retired January 2024); 6 Mary’s House for Older Adults, Inc., Washington, DC, USA; 7 Dana-Farber Cancer Institute, South Weymouth, MA, USA; 8 Brookdale Center for Healthy Aging, Hunter College, CUNY, New York, NY, USA; 9 Department of Internal Medicine, University of Colorado School of Medicine, Aurora, CO, USA; 10 Division of Palliative Medicine, University of Texas Medical Branch at Galveston, Galveston, TX, USA; 11 Brookdale Department of Geriatrics and Palliative Medicine, Icahn School of Medicine at Mount Sinai, New York, NY, USA

**Keywords:** LBGTQ+, serious illness care, palliative care, discrimination, transgender

## Abstract

**Objectives:**

Recent increases in homophobic and transphobic harassment, hate crimes, anti-lesbian, gay, bisexual, transgender, gender nonconforming, and queer (LGBTQ+) legislation, and discrimination in healthcare toward LGBTQ+ persons require urgent attention.

This study describes seriously ill LGBTQ+ patients’ and partners’ experiences of discriminatory care delivered by healthcare providers.

**Methods:**

Qualitative data from a mixed-methods study using an online survey were analyzed using a grounded theory approach. Seriously ill LGBTQ+ persons, their spouses/partners and widows were recruited from a wide range of organizations serving the LGBTQ+ community. Respondents were asked to describe instances where they felt they received poor care from a healthcare provider because they were LGBTQ+.

**Results:**

Six main themes emerged: (1) disrespectful care; (2) inadequate care; (3) abusive care; (4) discriminatory care toward persons who identify as transgender; (5) discriminatory behaviors toward partners; and (6) intersectional discrimination. The findings provide evidence that some LGBTQ+ patients receive poor care at a vulnerable time in their lives. Transgender patients experience unique forms of discrimination that disregard or belittle their identity.

**Significance of Results:**

Professional associations, accrediting bodies, and healthcare organizations should set standards for nondiscriminatory, respectful, competent, safe and affirming care for LGBTQ+ patients. Healthcare organizations should implement mechanisms for identifying problems and ensuring nondiscrimination in services and employment; safety for patients and staff; strategies for outreach and marketing to the LGBTQ+ community, and ongoing staff training to ensure high quality care for LGBTQ+ patients, partners, families, and friends. Policy actions are needed to combat discrimination and disparities in healthcare, including passage of the Equality Act by Congress.

## Introduction

Public attitudes toward the lesbian, gay, bisexual, transgender, gender nonconforming, and queer (LGBTQ+) community are at a turning point in the US and globally. The past decade has witnessed an array of social, cultural, legal, and political advancements affecting this community. Foremost of these has been the legal recognition and growth of same-sex marriages and expanded civil rights protections by the US Supreme Court (Bostock v. Clayton County, GA 2020; Obergefell v. Hodges 2015), continued expansion of LGBTQ+ civil rights protections in 22 states and the District of Columbia (Movement Advancement Project 2023), and greater acceptance by the wider culture (GLAAD 2023).

The backlash in conservative corners of the US has been severe, with increases in harassment, hate crimes, and discrimination seen nationally, primarily in conservative-leaning areas. The American Civil Liberties Union reports: 530 anti-LGBTQ+ bills introduced in 2024 (as of 9/5/24); 112 addressing healthcare restrictions; 44 bills became law, 10 of which involved healthcare restrictions. The Anti-Defamation League and Gay and Lesbian Alliance Against Defamation (GLAAD) report at least 356 incidents of hate crimes from June 2022 to April 2023 (ADL 2023). Anti-LGBTQ+ policies aim to restrict or deny access to gender-affirming care and stigmatize LGBTQ+ people without considering community concerns (Durso 2017; Fredriksen-Goldsen 2016; Medina and Mahowald 2023). These actions have led the Human Rights Campaign to declare a “national state of emergency for LGBTQ+ Americans”(Human Rights Campaign 2023).

There is growing evidence of discriminatory actions in healthcare received by the LGBTQ+ community. Our prior research found high levels of disrespectful and inadequate care due to the patient’s sexual orientation or gender identity, especially for Black and Hispanic patients and those living in politically conservative regions (Stein et al. 2023, 2020). LGBTQ+ patients fear discrimination, social isolation, and undignified death, need for social support, institutional safety, economic and legal supports, and advocacy to decrease barriers to care (Rosa et al. 2023). One study found that LGBT adults were more likely than non-LGBT adults to report negative healthcare experiences in the prior 3 years that caused their health to get worse, made them less likely to seek health care, or caused them to change providers (Montero et al. 2024). The majority reported preparing for insults from providers or staff. This discrimination increased anxiety, depression, and loneliness. Finally, a recent study found that LGBTQI+ individuals experienced disrespect and discrimination from their healthcare providers at much higher rates than non-LGBTQI+ individuals; these individuals were also more likely to delay or avoid necessary medical care in the prior year because of disrespect or discrimination (Medina and Mahowald, 2023). These reports were higher among LGBTQI+ respondents of color, with disability, or who identified as transgender, gender nonbinary, or intersex (Medina and Mahowald 2023; Stein 2020). This study aims to describe the experience of disrespectful and discriminatory care received by seriously ill LGBTQ+ patients and their partners from healthcare providers.

## Methods

### Study design

A mixed-methods study using a cross-sectional design was conducted. Respondents completed an online survey that took about 15 minutes to complete. This study was approved by the Institutional Review Boards at Yeshiva University (IRB #1303817) and Fordham University (IRB #1830).

### Sample

LGBTQ+ persons with a serious illness and their spouses, partners (either married or not), and widows were recruited between October 2021 and January 2023 (*n* = 290) from a wide range of organizations that serve the LGBTQ+ community, including healthcare organizations and medical centers, hospices and palliative care programs, and national and local organizations that serve this population. The study flyer was posted on organizational websites, social media, newsletters, and virtual and physical bulletin boards of these organizations. Sample characteristics are in a prior publication (Stein 2023).

### Measures

Respondents were asked 2 sets of closed-ended questions about 11 types of disrespectful and inadequate care from a healthcare professional or support staff that were experienced by the patient due to being LGBTQ+. They were also asked about 5 types of discriminatory care experienced by the partner due to the patient being LGBTQ+. Those findings have been previously reported (Stein et al. 2023).

The responses from the open-ended questions provided data for this report. Respondents were asked to describe poor care received from a healthcare provider because the person with a serious illness is LGBTQ+.

### Data analysis plan

A grounded theory approach with constant comparison analysis was used to code the data (Charmaz 2014). The first 2 authors coded the responses together using in vivo coding with Atlas.ti. They then jointly reviewed the first level codes to reduce the number of codes and eliminate redundancy. These first level codes were then sorted into higher level categories, which were then combined into the final categories presented here.

## Results

Six main themes emerged from the qualitative data: disrespectful care; inadequate care; abusive care; discriminatory care toward persons who identify as transgender; discriminatory behaviors toward partners; and intersectional discrimination. The quotes below demonstrating these themes are verbatim and were not modified by the authors. The sexual orientation or gender identity reported by respondents is indicated after each quote to provide context.

### Disrespectful care

Disrespectful care due to the patient being LGBTQ+ was reported in a wide variety of forms. Many participants reported being treated differently. Experiences ranged from explicit acts of incivility to broader interactions characterized by discomfort.

*Staff often act awkwardly around me once they learn my sexual orientation. There haven’t been any major incidents, but it’s enough to make medical care more stressful for me, which I don’t need.* [Lesbian respondent]

*It’s hard enough to receive adequate healthcare for a chronic or severe illness, and having to go through these experiences makes the process even more dehumanizing.* [Queer respondent]

*RN in pre-cancer surgery screen was so rude and insensitive during the visit it was the only time my wife cried from the time she was dx*[diagnosed] *to completed tx*[treatment]. [Lesbian respondent]

Respondents shared that these experiences eroded their sense of safety and caused some to hide their sexual orientation and relationship with their partner.

*My wife said she was treated differently when I was there. Felt it was best to present myself as a friend.* [Lesbian respondent]

*We were very quiet about our relationship. Afraid the staff would be unkind to her.* [Lesbian respondent]

Respondents felt disrespected by staff who imposed their conservative religious beliefs on them.

*Had a CNA wake my wife up in the middle of the night before her heart surgery to pray with her and have her turn away from being gay because if she died the CNA didn’t want her to go to hell.* [Lesbian respondent]

*Both of us have had medical professionals [who] had anti-LGBTQ+ religious beliefs shared during appointments.* [Gay respondent]

*A doctor proselytized to me about the sin of being queer.* [Transgender]

*Medical professional had anti-LGBTQ+ religious beliefs they shared.* [Lesbian respondent]

There were reports of relationships with partners not being acknowledged, understood or respected. Legal marriage was also disregarded as well as other intimate partnerships.

*I was asked about my marital status and I responded “widowed.” The response was “now how can you be widowed?”, as though my relationship didn’t rise to the same level as that of a heterosexual married couple.* [Gay respondent]

*A doctor who I had been seeing for several months expressing shock to learn I had a same-sex partner – the same doctor treating my partner rudely and unprofessionally.* [Queer respondent]

*Sitting in my hospital bed with my boyfriend (we are of different races/ethnicities) after a serious surgery and being asked with a smirk if he was my brother.* [Queer respondent]

*My partner’s gender was not recognized by staff, and staff assumed we were either a heterosexual couple, or that my partner was my parent.* [Lesbian respondent]

*Not overtly rude, just not acknowledged in the same way as straight people losing spouses.* [Lesbian respondent]

Some patients and partners chose to hide their sexual orientation and relationship to avoid disrespect and discrimination. Some respondents mentioned that they did not feel safe revealing their sexual orientation.

*We were very quiet about our relationship. Afraid the staff would be unkind.* [Lesbian respondent]

*I’ve never disclosed my LGBT+ identities because I’m in a rural area and they have done nothing to indicate that it might be safe to do so.* [Queer respondent]

*We have the conversation, if we are in certain places, do we tell them that we are lesbian?* [Lesbian respondent]

*I’ve realized that my own concerns and worries about the possibility of being treated differently have sometimes led me to not be totally open about my sexual orientation and relationship status. I’ve sometimes withheld or delayed discussions until I felt comfortable disclosing it.* [Gay respondent]

*At my partner’s day program, they feel as if they cannot say what is on their mind for fear of being made fun of by the other members and the staff (*social workers). [Transgender respondent]

One respondent commented on outdated forms that do not include LGBTQ+ identities.

*Intake forms have been a big source of either not feeling seen at all/feeling fully excluded or feeling welcomed and heard before I even met the provider. I have come across terrible forms even in LGBTQIA+ friendly clinics. It’s amazing to me how much the tiniest details matter and how often they are overlooked.* [Transgender respondent]

### Inadequate care

There were many reports of inadequate care that respondents attributed to being LGBTQ+. This lack of appropriate care was experienced in myriad ways, including undertreatment, mistreatment, and poor quality care delivery.

*They would look at me differently and not treat me very well.* [Lesbian respondent]

*He [*doctor] *also neglected to schedule me for regular screening. Had I not fired him for being transphobic and my new doctor not scheduled an MRI, the mets in my lung and peritoneum would have been missed and I would be dead by now.* [Transgender respondent]

*I have had so many poor experiences with healthcare providers because I am LGBTQ+.* [Lesbian respondent]

Some respondents reported feeling ignored by professional and support staff.

*She was frequently ignored – nurses and aides did not check in with her in her room, no one answered the call button when she needed to use the bathroom, no one offered to help her wash up, no one brought in her food tray for hours even though she repeatedly asked.* [Lesbian respondent]

Some respondents reported that their concerns about inadequate care were dismissed by providers.

*When I said to the hospice coordinator that we will need assurance that the home health aide will be trained in working with LGBTQ+ patients, the coordinator said, “I’ll tell them.” The supervisor said I could assume any healthcare aide would be sensitive to our needs as LGBTQ+ clients.* [Lesbian respondent]

Another serious form of inadequate care is being denied treatment.

*Dr refused to treat me due to false ideas, so i went to another Dr.* [Gay respondent]

*I went to an endocrinologist to inquire about testing for PCOS* [Polycystic Ovary Syndrome]*. Upon the doctor entering the room, she asked “What are you doing here?” and before I could complete my answer, she added, “We don’t do trans!”* [Queer respondent]

### Abusive care

There were fewer reports of abusive care than other types of discriminatory care, but they are important and disturbing. The most common type of abusive care reported was withholding pain medication.

*Doctors have purposefully withheld pain medications.* [Lesbian respondent]

*She would beg for some pain meds but staff wouldn’t respond.* [Lesbian respondent]

*I was laughed at when spasming out due to lack of pain medication and threatened by the head nurse when my partner called to discuss my treatment.* [Transgender respondent]

### Discriminatory care toward persons who identify as transgender

There were many reports of disrespectful care from respondents who identified as transgender.

*I have come to greatly fear how I will be treated because of being transsexual. I never know how they will react or how I will be treated.* [Transgender respondent]

*He [*doctor*] made a big deal of telling me that I was a female and would always be a female medically. He was very insensitive and rude.* [Transgender respondent]

*Most of the time, we did NOT discuss the fact that I am trans with my spouse’s caregivers, out of concern that this would interfere with her care.* [Transgender respondent]

Respondents identifying as transgender also reported inadequate care.

*I felt that my pain and suffering was underplayed because they saw me as mentally ill because I was dressed in women’s clothing.* [Transgender respondent]

*The doctor provided useless, substandard care. I never mentioned being genderqueer to a doctor’s office ever again.*[Transgender respondent]

A transgender patient reported delaying care due to fears about transphobic healthcare providers.

*Once medical staff find out that I am transsexual*, *I never know how they will react or how I will be treated. ]I often have waited too long to receive care as a result of my fears so it definitely affects the outcomes of my medical situation.* [Transgender respondent]

A very common form of disrespectful care was misgendering and using the incorrect name and pronouns.

*I have been blatantly disrespected and misgendered by doctors despite notifying my care team of name and pronouns, and I feel as if I was not being taken as seriously.* [Transgender respondent]

*I was misgendered by my former GI oncologist on each visit.* [Transgender respondent]

*They are constantly misgendered and although their pronouns are clearly presented in the database, nurses, doctors and counselors use their dead name and incorrect pronouns which turns my partner away and makes them feel invisible.* [Transgender respondent]

*I’ve been called “it” and “he” by many healthcare professionals.* [Transgender respondent]

*The doctor himself ignored my repeated asking to be referred to in gender neutral terms.* [Gender nonbinary respondent]

### Discriminatory behaviors toward partners

In addition to disrespectful and inadequate care experienced by seriously ill LGBTQ+ persons, partners were also discriminated against. Some healthcare providers did not acknowledge the relationship between the patient and partner and/or were disrespectful to the partner.

*Not being acknowledged by a doctor as the spouse even after introduction as such.* [Lesbian respondent]

*Having to explain who I am when it is in the record that I am the SPOUSE.* [Lesbian respondent]

*My spouse is often treated with disrespect, experiences microaggressions related to her gender expression.* [Lesbian respondent]

One way in which this occurred was limiting the partner’s access to the patient.

*I was denied entry to the ICU to see my partner. I took him to the hospital and showed them my Medical Power of Attorney for him.* [Gay respondent]

*One time, my mother had to sneak my partner into the hospital to see me. If my mother had been opposed to having her there, she would not have been allowed access to me.* [Lesbian respondent]

*My partner was barred from the ER and not allowed to speak with the MD to give further information when I wasn’t able to speak for myself.* [Transgender respondent]

Some respondents reported that their partner’s role as legally-appointed surrogate was disregarded.

*My wife was advocating for me for the poor care I was receiving in the hospital. The nurse yelled at my wife and told her, while I was present, that “I hope they kick you out.”* [Lesbian respondent]

*She has been told she has to wait in the waiting room instead of accompanying me in for procedures when other spouses are.* [Lesbian respondent]

In some situations, healthcare providers deferred to family of origin even though the partner was the legal surrogate.

*My partner’s relationship with her family of origin was labile. Yet, repeatedly, hospitalists choose to speak to them rather than me. Often I had to aggressively assert my rights as partner’s advocate.* [Lesbian respondent]

*Partner was in ER on vent. I was told to leave as only family was permitted. This was despite healthcare directive being on file at hospital. My questions and concerns were ignored.* [Lesbian respondent]

### Intersectional discrimination

Respondents spoke of discriminatory care due to identifying as LGBTQ+ and as a member of other groups that experience discrimination. Some respondents mentioned race as a factor in receiving disrespectful or inadequate care.

*Not treated in the same way as others I saw. Compassion for losing a spouse. My children (young adults) were not treated as though they were losing their mother. My children are black, my wife’s white family were acknowledged as grieving.* [Lesbian respondent]

*Obviously a Black man and once they are informed I am gay/homosexual, they question my statement because “I don’t look gay.”* [Gay respondent]

Some respondents mentioned other conditions they felt contributed to discrimination because they were LGBTQ+.

*When you combine being a queer trans (nonbinary) person with being neurodiverse and obese, my medical treatment and the prejudice I face in the medical community is worse than it would be.* [Gender nonbinary respondent]

*it was difficult to get any provider to listen to me, though I think in some cases that was related to my transness/queerness and other times was related to mental health stigma.* [Transgender respondent]

## Discussion

This study provided rich qualitative data on seriously ill LGBTQ+ patients’ and partners’ experiences of disrespectful, discriminatory, and poor quality care during very vulnerable times in their lives. In conjunction with the quantitative findings from this study (Stein et al. 2023) and our survey of healthcare providers (Berkman et al. 2023, 2024; Stein et al. 2020), this study provides further evidence that LGBTQ+ patients experience a concerning level of disrespect and discrimination from healthcare providers, even when they are seriously ill. Transgender patients relay unique forms of discrimination that disregard or belittle their identity. These findings are consistent with recent studies (Medina and Mahowald 2023; Montero 2024).

### Discriminatory care

Disrespectful care is described by patients as being treated worse than non-LGBTQ+ patients, including having religious beliefs imposed on them, feeling unsafe to reveal their sexual orientation, and medical forms that exclude their identities. Staff may fail to acknowledge, respect, and include partners in important health decisions. While disrespectful care might be viewed as less serious than inadequate or abusive care, it may negatively affect the trust of patients in healthcare providers, cause them to delay or avoid necessary care, or not disclose their sexual orientation or gender identity – information necessary to provide quality health care (Medina and Mahowald 2023; Montero et al. 2024). Patients may hide their partner at a time they most need them as surrogate decision-makers and to provide emotional support.

### Care to persons who identify as transgender

Transgender communities have been targeted in conservative states by legislation aimed at restricting access to gender-affirming care. Reports abound of individuals and families moving to more progressive states, both for healthcare and a more accepting environment, often at economic and social cost (DePillis 2024). These societal trends affect serious illness care, as evidenced by respondents’ reports of belittling, disrespect and discrimination by healthcare providers, often through misgendering and using incorrect pronouns (Berkman 2024; Medina and Mahowald 2023; Montero et al. 2024; Stein et al. 2023, 2020).

### Implications for practice and policy

There are many policy actions that would combat discrimination and disparities in healthcare. Congress should pass the Equality Act to provide comprehensive civil rights standards in all areas of daily life, including healthcare. This would create a national standard, applicable to states with few such protections and anti-LGBTQ+ laws. Executive Orders and federal regulations, including those of the Department of Health and Human Services, should continue to support the rights to health care and personal autonomy (Centers for Medicare and Medicaid Services 2019; Executive Order of the President 2021; Patient Protection and Affordable Care Act 2010). State law is also needed to protect LGBTQ+ people from discrimination.

Professional associations and healthcare organizations should set standards for nondiscriminatory, respectful, and affirming care for LGBTQ+ people. For example, the National Hospice and Palliative Care Organization issued an *LGBTQ+ Resource Guide* to address LGBTQ+ healthcare disparities and provide guidance for community outreach (National Hospice and Palliative Care Organization 2021).

Healthcare organizations should consider a multipronged approach, including: mechanisms for identifying problems and ensuring nondiscrimination in services and employment; strategies for outreach and marketing to the LGBTQ+ community, especially in states with punitive anti-LGBTQ+ laws; and mandatory ongoing training for professional and support staff to ensure safe, respectful and competent care for LGBTQ+ patients, partners, families, and friends. Staff training should include inquiring about sexual orientation, gender identity, and important relationships in a nonjudgmental and routine manner (Acquaviva 2023). Special efforts are needed to counter the political and cultural environment that stigmatizes the trans community.

### Strengths and limitations

Our survey is one of the first to explicitly ask about the experiences of seriously ill LGBTQ+ patients and their partners. The sample was relatively large and was recruited from many organizations and social media outlets, from national to local sites. The sample was diverse by sexual orientation, gender identity, age, ethnicity, and region of the US (Stein et al. 2023). There was selection bias due to recruiting respondents mainly through online methods that required access and ability to complete an online survey. Residents of long-term care facilities were not included.

Another concern is that respondents included only persons, or their partners, identifying as LGBTQ+. Without a comparison group, it is difficult to be sure that the poor care reported was actually due to this reason. However, both the Kaiser Family Foundation (KFF) and Center for American Progress studies have included non-LGBTQ+ control groups and reported findings that affirm our findings (Medina and Mahowald 2023; Montero et al. 2024).

### Future research

Future studies would benefit from greater representation of persons of color, from rural areas, and with limited incomes. Including residents of long-term care facilities is important due to concerns about poor care in some of these facilities (Rau 2024; Stein et al. 2010; Sugathapala et al. 2023; Travers et al. 2023). Conducting interviews and focus groups would allow for probing responses and obtaining more details about poor care experienced by this population. Research should inform best practices to improve care.
